# Extracellular Vesicles: A Novel Target for Exercise-Mediated Reductions in Type 2 Diabetes and Cardiovascular Disease Risk

**DOI:** 10.1155/2018/7807245

**Published:** 2018-06-19

**Authors:** Natalie Z. M. Eichner, Uta Erdbrügger, Steven K. Malin

**Affiliations:** ^1^Department of Kinesiology, University of Virginia, Charlottesville, VA, USA; ^2^Division of Nephrology, University of Virginia, Charlottesville, VA, USA; ^3^Division of Endocrinology and Metabolism, University of Virginia, Charlottesville, VA, USA; ^4^Robert M. Berne Cardiovascular Research Center, University of Virginia, Charlottesville, VA, USA

## Abstract

Regular exercise is important for reducing type 2 diabetes (T2D) and/or cardiovascular disease (CVD) risk. However, only about 40–50% of this CVD risk reduction is accounted for by adiposity, hyperglycemia, hypertension, and dyslipidemia. Herein, we present the novel hypothesis that extracellular vesicles (EVs) are candidate biomarkers that may relate to impaired endothelial function and insulin resistance independent of obesity risk factors. EVs are small membrane-bound particles that are generated by cells following stimulation, stress, or activation. They carry markers of their parent cell and are thought to be potent bioactivators and communicators. We discuss the underlying physiology of specific cell type EVs, as well as examine how acute and chronic exercise interventions impact EV count and phenotype. We also propose that current gaps in the field are in part related to use of different detection techniques and the lack of standardized measurements of EV affecting the pre- and postanalytical phase. Ultimately, improving the understanding of how EVs impact cardiometabolic health and their function will lead to improved approaches for enhancing diagnostic options as well as designing exercise interventions that treat and/or prevent T2D and CVD.

## 1. Introduction

Nearly 33% of all deaths globally each year are attributed to cardiovascular disease (CVD) [1]. In fact, CVD mortality has increased from 12.59 to 17.82 million between 1990 and 2015 [2]. Individuals with type 2 diabetes (T2D) are 2-3 times more likely to have CVD than their healthy counterparts, indicating that abnormalities in glucose metabolism share a CVD pathogenic root [3]. However, glucose alone may not be a primary driver of CVD in people with T2D given recent interventions focused on lowering glucose alone have failed to significantly lower CVD risk and mortality [4]. As such, it is not surprising that elevated blood pressure and dyslipidemia in people with hyperglycemia are considered critical drivers of CVD that are linked together by insulin resistance [5]. Insulin resistance can be defined as the reduced responsiveness of skeletal muscle, liver, adipose, and vasculature to insulin for the maintenance of nutrient delivery and utilization. Although the exact cause of insulin resistance is unclear, endothelial dysfunction is a leading candidate for promoting these nutrient disturbances [6]. Endothelial function is the ability of the endothelium to respond to both metabolic mediators (e.g., insulin and nitric oxide) and/or shear stress that enhance blood flow. Recently, the American Heart Association suggested that current biomarkers (e.g., blood pressure and lipids) do not account for the majority of adverse outcomes and may account for only 40–50% of CVD risk [7]. Thus, there is an urgent need to identify new treatment targets for T2D and CVD that mediate health and well-being.

Extracellular vesicles (EVs) have emerged as novel biomarkers of T2D and CVD [8, 9]. EVs belong to a heterogeneous population of vesicles summarized with the generic term “Extracellular Vesicles” (EVs). Interestingly, most of the studies analyzing EVs in metabolic diseases have focused on larger EVs (>100–1000 nm) (Tables 1 and 2, resp.), called microparticles/microvesicles, generated by the low centrifugation speed of up to 20,000 Gs, but our own data [10] and that of others [11–13] indicate that we also see smaller EVs (<100 nm, called exosomes) in these EV preps. As these studies have likely analyzed a mix of larger and smaller EVs of different densities, we will only use the term EV. EVs are unique biomarkers as they are also believed to carry and transfer proteins, lipids, and nucleic acids, and they facilitate communication between cells. How EVs regulate vascular health remains to be fully determined, but obesity-related insulin resistance might be a potential reason through oxidative stress and inflammatory-related mechanisms [14]. Interestingly, physical inactivity also increases EV levels in association with worsening of insulin resistance and endothelial dysfunction, suggesting that muscle contraction alters disease risk in an EV-mediated manner [15]. However, there is limited research regarding the effects of physical activity and/or exercise on EVs in healthy and disease populations. In particular, we propose that EVs may be a novel mediator of T2D and CVD risk. First, we highlight the biogenesis of EVs and the purported mechanism relating EV to insulin resistance and endothelial function. Next, we examine the gaps in knowledge regarding the effectiveness of acute and chronic exercise on EVs. We also discuss the mechanistic role of cell-specific EVs related to leukocytes, platelets, and the endothelium as mediators of cardiometabolic risk. Lastly, we analyze how current EV methodologies could play a role in discrepancies seen across exercise studies and discuss new methodology to advance understanding of EVs and exosomes that could improve diagnostic and treatment options for T2D and CVD.

## 2. Extracellular Vesicle Biogenesis

First described as merely “cell dust” by Wolf in 1967, EVs are now recognized as cell bioactivators and communicators of cardiometabolic health [16]. Smaller EVs (<100 nm, also called exosomes) are thought to derive from multivesicular bodies inside the cells that are then secreted into different body fluids, whereas larger EVs (>100–100 nm, also called microparticles/microvesicles) are believed to be shed from cells into body fluids/tissue upon stimulation or activation. These larger EVs are likely the product of outward membrane budding through cytoskeletal rearrangement and a loss of calcium-dependent membrane phospholipid asymmetry [17]. These vesicles consist of membrane proteins and cytosolic material from the cell they originate from. Indeed, EVs are derived from cells in circulation (i.e., endothelial, platelet, and leukocyte), erythrocytes [18], as well as progenitor cell populations [19] (Table 3). Additionally, EVs are found in many other body fluids besides blood, including urine [20], which increase the potential for clinical collection sites. EVs are released during conditions of stress that initiate cell activation and/or apoptosis [21]. In particular, proinflammatory stimuli (e.g., oxidative stress/cytokines), bioactive lipids [22], and hyperglycemia [23] are considered key stimuli that impact EV release, phenotype, and function. In particular, hyperglycemia increases endothelium-derived EV formation, size, and reduces surface charge that collectively prompt greater procoagulant activity [24]. Moreover, high glucose conditions increase NADPH oxidase activity in endothelial EVs that work to amplify the effects of oxidative stress-mediated inflammation on the endothelium [23] that decrease endothelial nitric oxide synthase (eNOS) [25], thereby potentially impairing vascular function and raising CVD risk. There is also work suggesting that EVs may not only release inflammatory cytokines [26] but also act as deliverers of bioactive lipids [22], protein, and genetic material [10] between cells. Taken together, EVs represent a potentially novel paradigm in cell-to-cell communications between various organs important for T2D and CVD. For a comprehensive discussion of biogenesis of EVs, we will refer the reader to other review papers [27].

## 3. Extracellular Vesicles in The Pathogenesis of T2D and CVD

EVs are composed of parental proteins, nucleic acids, and cytoplasm based on the stimuli [10]. This is physiologically important because carrying markers of the parent cell allows for specific subpopulation identification (e.g., endothelium- or leukocyte-derived) [10] that can influence crosstalk between tissues and cells [28]. Indeed, elevated endothelial EVs are thought to reflect vascular injury, whereas increased leukocyte and platelet EVs signify proinflammation and coagulation, respectively. This notion is consistent with literature reporting that different subtypes of EVs are elevated in people with prediabetes [29], T2D, and CVD [8, 9] as well as hypertension [30], chronic kidney disease [31], and heart failure [32]. Even obesity, independent of comorbidities, presents with elevated platelet EV levels [33] in relation to reduced fibrinolytic ability. Subsequently, these observations support that EVs likely play a key physiologic role above and beyond a biomarker.

**Figure 1:**
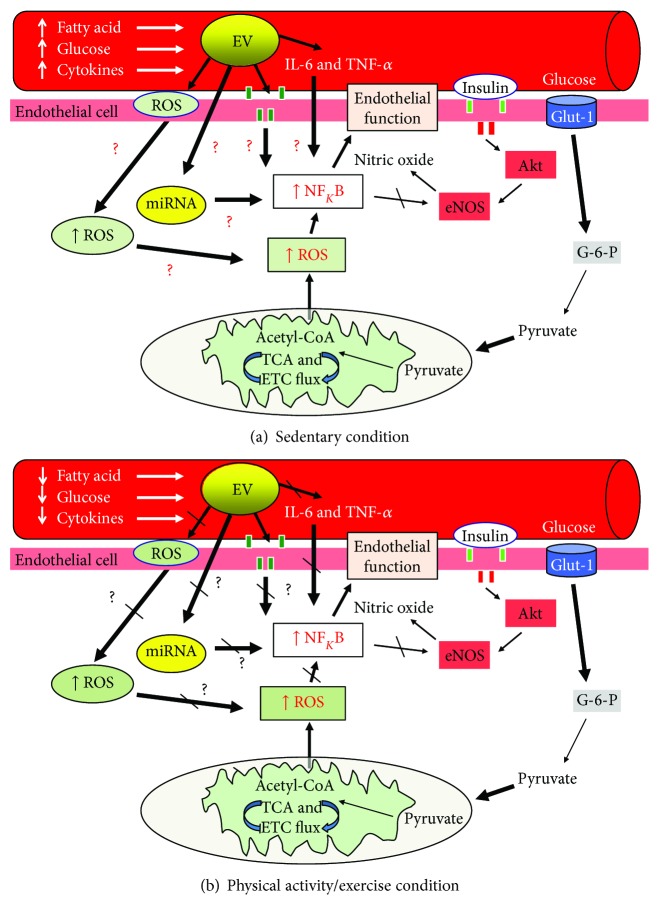
Working hypothesis by which extracellular vesicles (EVs) interact with exercise to influence vascular function and insulin sensitivity. Reactive oxygen species (ROS) are generated by EVs in response to bioactive lipids, glucose, and inflammatory cytokines and act as important cellular regulators in cell health. In addition, EVs may bind to cells and interfere with receptor-related mechanisms and/or release microRNA (miRNA) to influence cell activity. Lastly, EVs may release inflammatory cytokines and impact cell NF*κ*B activity, which influences cell vascular function. Exercise (b) decreases circulatory lipids, glucose, and cytokines, thereby improving EV levels and function. We hypothesize herein that EVs not only serve as a biomarker of type 2 diabetes and cardiovascular disease but also regulate vascular function independent of traditional obesity-related risk factors. Future work should consider studying the interaction of EV and exercise doses in order to identify optimal treatment plans for preventing type 2 diabetes and cardiovascular disease.

Circulating EVs are believed to play an important physiologic role in vascular physiology [34] (Figure 1(a)). Werner et al. reported that elevated endothelial EVs (CD31^+^/Annexin V^+^) are correlated with reduced endothelium-dependent vasorelaxation [35]. This is consistent with others reporting that elevations in these same endothelial EVs are related to reduced flow-mediated dilation as well as increased pulse wave velocity and carotid intima-media thickness [36, 37]. Together, these findings suggest that higher levels of EVs relate to poor blood flow and arterial stiffness. There are several putative mechanisms that may explain how EVs promote dysregulation of blood flow, although most data exists from *in vitro* experiments and more human work is needed. EVs are thought to directly produce reactive oxygen species (ROS). Endothelial EVs (CD144, Annexin V^+ve^) increase production of superoxide anion and hydrogen peroxide in cultured endothelial cells through NADPH oxidase and mitochondria [38, 39], although others suggest that xanthine oxidase may contribute in endothelial (CD144-PE) [40], lymphocytic (CD4, CD3^+^, CD8, CD11a, Fas, and FasL) [41], and monocyte-derived EVs [42]. Additionally, EVs are hypothesized to promote *in vivo* inflammation through stimulation of proinflammatory cytokines and the recruitment of inflammatory cells [26]. In *in vitro* experiments, leukocyte EVs (CD14^+^) promote the release of IL-6 and IL-8 in cultured endothelial cells [43]. In addition, T-cell EVs promote TNF-*α* and IL-1b by monocytes [44] and promote the interaction and adhesion of leukocytes to endothelial cells [22]. These later findings are consistent with work by Mastronardi et al. [45] demonstrating that injection of EVs from blood of patients with sepsis into mice promotes increased expression of iNOS, COX-2, and NF*κ*B in the heart and lung, thereby supporting a direct role of EVs at producing inflammation. Lastly, circulating EVs express the functionally active eNOS protein [25]. This is clinically germane as patients with endothelial dysfunction have EVs with reduced expression and release of nitric oxide [25].

Another possible mechanism by which EVs contribute to T2D and CVD relates to the interaction and transfer of EV contents to the cell (Figure 1(a)). EVs have been proposed to physically alter cell target receptors that modify signal transmission. For example, blocking EGF receptors in endothelial cells inhibits EV-mediated ROS production and inflammation [38]. Additionally, other work has suggested that EVs from obese subjects reduce insulin-stimulated glucose uptake [46] and macrophage-derived EVs (M0 THP-1) interfere with GLUT-4 translocation in human adipocytes by decreasing p-Akt, thereby inducing insulin resistance [47]. The exact cause of this insulin resistance remains to be elucidated, but activation of NF*κ*B was noted, suggesting that inflammation may play a role. Indeed, it is also possible that miRNA transcripts from EVs also play an important role in communicating signals to local and systemic tissues for the alteration of cell activity [48]. For instance, Rautou et al. [18] demonstrated that EVs (CD31^+^) derived from apoptotic plaques transferred ICAM-1 to endothelial cells, suggesting that EVs play an important inflammatory response mechanism in atherosclerosis. In addition to ICAM-1, other studies have reported adipocyte-derived EVs (CD14^+^) to interfere with insulin signaling in both the liver [49] and skeletal muscle [50] via transfer of adipokine content, thereby inducing insulin resistance [46]. However, not all studies support the observation that EVs fuse and transfer content to cells [51], as there are different ways in which EVs promote cell-to-cell communication or even EV uptake [52]. In either case, EVs appear to mediate angiogenesis and induce endothelial repair [34, 53–55] by at least partially [56] vascular endothelial growth factor-A [57] or eNOS [25]. In this way, EVs may promote increased angiogenesis and blood flow via cargo such as eNOS-induced nitric oxide. In turn, this compensatory response of increased blood flow may allow nutrient delivery to tissue, thereby contributing to insulin-mediated GLUT-4 translocation. Given the literature linking oxidative stress and inflammation to the pathogenesis of insulin resistance and endothelial dysfunction [58], the identification of how EVs may be modified or targeted for metabolic health warrants attention.

## 4. Effects of Acute Exercise Bouts on Extracellular Vesicles

A majority of the chronic exercise training induced an effect on insulin resistance and endothelial function is considered to be the result of the last bout of exercise [59]. Subsequently, understanding the acute exercise effect on EVs provides insight independent of cardiorespiratory fitness adaptation and weight/fat loss. However, to date there are limited studies examining the effects of acute aerobic [60, 61] or resistance exercise [62] on EVs (Table 1). For instance, Mobius-Winkler et al. tested the effect of a 4 hr cycling protocol at 70% of the anaerobic threshold in 18 young, lean, healthy males [63] and found no change in endothelial EVs (CD42b^−^, CD42b^−^/CD62E^+^) in the immediate postexercise period, despite increases in cytokine IL-6. It was speculated that the lack of exercise effect might have been due to the population studied (healthy versus diseased) or the low to moderate intensity exercise prescribed. We add to this by speculating that the lack of EV differences following exercise could also be related to technical differences of EV detection. Blood was collected and EV pellet was enriched from platelet-poor plasma using conventional flow cytometry. Smaller EVs (e.g., <500 nm) might not have also been captured with this approach. In addition, targeted phenotyping was limited to detection of the surface marker for CD62E and CD42. CD62E is found on activated endothelium, but other endothelial markers might reflect better the endothelial changes during exercise. Nonetheless, these findings are consistent with Guiraud et al. who showed that there was no change in endothelial EVs (CD31^+^, CD62E^+^, and CD42b^−^) or platelet EVs (CD42b^+^) in 19 male coronary heart disease patients when measured up to 72 hr following either high-intensity interval or moderate-intensity cycling exercise [60]. In contrast, Chanda et al. reported that a maximal bout of exercise (defined as a VO_2_max test) elicited an approximate 40% increase in platelet (CD41a) EVs in healthy adults [61]. While these later findings suggest that exercise intensity raises EV, it should be noted that maximal exercise would be considered a stressful perturbation to the system and it is known that high-intensity exercise raises oxidative stress and inflammation in the immediate postexercise period, thereby conferring a stimulus for metabolic adaptation [64]. Indeed, *in vitro* experiments by Wilhelm et al. demonstrate that EVs generated after intense exercise in healthy young men enhanced endothelial proliferation, migration, and tubule formation compared with EV derived from the rest [65]. Interestingly, platelet EVs (CD41^+^) from the same patients were elevated during 1 hr of high (67% VO_2_max), but not moderate (46% VO_2_max), intensity exercise. These later findings are of potential significance as they suggest that exercise intensity promotes angiogenesis for improved blood flow and nutrient delivery. Whether EVs from people with T2D or CVD respond to exercise comparably to lean healthy people remains to be seen. This is particularly of interest given recent work highlighting that single bouts of exercise increase EVs (ACTN4, ADAM10, ALIX, ANAX11, and CD81) and miRNA to potentially coordinate communication of nutrient homeostasis between muscle, endothelium, as well as liver [66, 67].

Another possible reason explaining why acute exercise has yielded equivocal EV results may relate to sex differences. Toth et al. reported elevated total Annexin V EV, platelet EV (CD63, P-selectin-exposing), and endothelial EV (CD62, E-selectin-exposing) in 27 young healthy women compared to men while at rest. It was reported that elevated EVs (Annexin V-binding EV, CD61, P-selectin-exposing EV, and E-selectin-exposing EV) in women were related to the menstrual luteal cycle [68]. However, no significant sex- or menstrual cycle-dependent differences were observed in the endothelial EV (CD144^+^). Lansford et al. [69] recently tested the effect of an acute bout of exercise (60–75% VO_2_max) on endothelial EVs (CD62E^+^, CD31^+^/CD42b^−^, CD34^+^) in recreationally active men and women and demonstrated that endothelial EV (CD62E^+^) increased by 107% in men but not in women. Conversely, women displayed a 253% elevation in mononuclear EVs (CD34^+^). Based on previous research [70, 71], these results suggest that increased levels may prime CD34^+^ peripheral blood mononuclear cells for paracrine angiogenic effects in females. Interestingly, the endothelial EVs (CD31^+^/CD42b^−^) remained unchanged following exercise in either sex. These results suggest that only certain phenotypes of endothelial EVs (CD62E^+^) or other not yet tested EV phenotypes may be affected by sex and exercise. In another study, Durrer et al. examined the effects of high-intensity continuous versus interval exercise on EVs in young, overweight inactive adults and reported that both exercises lowered EVs in men (*n* = 6), but endothelial EV counts (CD31^+^/CD42b^−^) were unaffected in females (*n* = 7) [72]. However, high-intensity continuous exercise increased endothelial EVs (CD62E^+^) in females. Although this was a relatively small sample size, the data suggest that sex may be an important factor explaining differential EV responses to exercise. Further work is needed to elucidate the mechanism by which men and women differ in EV profiles in order to individualize exercise to treat and/or prevent disease.

It reasons that dietary intake may also influence EV responses postexercise since circulating bioactive lipids are considered a stimulus for EV biogenesis. In fact, high-fat meals induce endothelial dysfunction in healthy and T2D individuals [73]. Although Jenkins et al. reported that a high-fat meal had no independent effect on endothelial EVs, acute exercise at 70% VO_2_max lowered endothelial EVs (CD62E^+^ and CD31^+^/CD42b^−^) by 55% and 30%, respectively (both *P* < 0.05) compared to a sedentary control in healthy, recreationally active men [74]. Interestingly, the lowering of endothelial EVs (CD62E^+^ and CD31^+^/CD42b^−^) postexercise was associated with blunted ROS production during postprandial lipemia. This finding supports the notion that EVs may induce vascular dysfunction through an oxidative stress-mediated mechanism. The modulation of oxidative stress postexercise may also be clinically relevant since it relates to fasting and postprandial endothelial dysfunction in obese individuals with prediabetes [75]. In addition, a lowering of endothelial EVs (CD31^+^, CD31^+^/CD42b^−^), which may be indicative of endothelial activation and apoptosis, suggests that exercise confers cardiovascular protection through modulation of the EV phenotype. However, Harrison et al. reported that high-intensity exercise performed at ~70% VO_2_max for 90 min had no effect on high-fat-fed-induced elevations in endothelial EVs (CD31^+^/CD42b^−^) in recreationally active young men [76]. This observation is in stark contrast to Jenkins et al. [74]. Despite both studies prescribing exercise at 70% VO_2_max, Harrison et al. included ten 1 min sprints. This subtle difference in exercise protocols may be of relevance since high-intensity exercise could have promoted greater vascular injury and prohibited the lowering of EVs. Additionally, differences in EV preparation and analysis, such as centrifugation at 1500*g* for 20 min at room temperature [74] as opposed to 1600*g* for 15 min at 4°C [76], may account for differences between the two studies. In either case, additional work is required to determine if exercise restores diet-induced EV levels to optimize exercise prescription for disease prevention in men and women given that postprandial metabolism is a strong predictor of CVD [77].

## 5. Effects of Chronic Exercise Training on Extracellular Vesicles

Exercise training improves whole body insulin sensitivity [78, 79] and glucose tolerance [80, 81] in adults with prediabetes and T2D. Additionally, chronic exercise enhances endothelial function in healthy individuals [82] and those at risk for [83] or with CVD [84, 85]. Therefore, it would be expected that long-term exercise training would also have favorable effects on EV phenotype and count. Bruyndonckx et al. recently demonstrated that 10 months of exercise training significantly decreased endothelial EVs (CD31^+^/CD42b^−^) as measured by conventional flow cytometry in 33 overweight children [86]. In addition to decreasing endothelial EVs, exercise training significantly improved microvascular function (measured via pulse amplitude tonometry), increased circulating adiponectin, and reduced body fat and high-sensitivity C-reactive protein. These findings are consistent with other work reporting that 12–24 weeks of aerobic exercise with weight loss significantly lowered endothelial EVs (CD31^+^/CD41a; CD62E^+^) in middle-aged men with erectile dysfunction [87] or prehypertensive men and women [88], as well as in African American women [89, 90] (Table 2). Interestingly, changes in endothelial EVs (CD62E^+^), IL-6, and IL-10 accounted for nearly 11% of the improvements in flow-mediated dilation following exercise training in the later studies [89, 90].

Although exercise training appears to favorably lower endothelial EVs (CD31^+^/CD41a; CD62E^+^), not all individuals appear to respond the same [91]. Kretzschmar et al. [89] demonstrated that endothelial EVs (CD31^+^/CD42b) only decreased in premenopausal compared with postmenopausal women following exercise training. It is not clear why postmenopausal women did not respond to exercise, but it is consistent with work suggesting some individuals are “exercise resistant” [75]. Another plausible reason may relate to the notion that estrogen provides protective heart effects and lowers CVD risk in women [93]. Notwithstanding these hormonal differences across the lifespan in women or compared with men, fitness may be an additional determinant of EV improvement posttraining. Indeed, recent work by our group [94], following minimal requirements of EV detection and functional studies established by the International Society of Extracellular Vesicles [12], as well as advanced imaging flow cytometry (*see below for details*), showed that EVs correlate with aerobic fitness and other cardiometabolic health factors in obese adults, highlighting again the potential role of fitness in modulating EVs. Furthermore, Van Craenenbroeck et al. reported that preintervention endothelial EVs (CD31^+^/CD42b) count significantly predicted improvements in VO_2_max despite no effect of a 12-week training program on these EVs in 200 individuals with coronary artery disease [95]. Together, these later findings suggest that EV may modulate training responses through a yet to be defined mechanism.

As exercise training promotes weight loss and decreases adipose-derived inflammation [96, 97], it is reasonable to expect that habitual exercise improves EVs originating from platelets and leukocytes. Murakami et al. reported that platelet EVs (CD41^+^) were significantly correlated with a subcutaneous fat area in 49 obese, nondiabetic subjects following 12 weeks of a restricted caloric diet or a restricted caloric diet plus exercise [33]. Although EVs did not correlate with visceral fat, which is considered a chief site for inflammatory production, this finding is reasonable since subcutaneous tissue is a primary supplier of free fatty acids, and elevated free fatty acids may act as a bioactive lipid that stimulates coagulation and platelet recruitment [98]. Whether exercise or exercise plus diet alter free fatty acid mediated EV levels or function waits to be tested. In either case, in the only studies to investigate exercise on leukocyte EVs (CD16^+^, CD14^+^), it was shown that training decreases neutrophil- and monocyte-derived EVs (CD16^+^, CD14^+^). This observation highlights that exercise has multicell EV effects that may favor improvement in cardiometabolic health [99, 100].

## 6. Extracellular Vesicle Analysis and Gaps

To date, most exercise studies lack sensitivity to optimally enrich and phenotype EVs. A leading challenge in doing so is the lack of consensus on the nomenclature of EVs as well as the precise detection method or sample preparation (i.e., the preanalytical phase) [12, 101]. In fact, the preanalytical phase includes several important steps that could impact the clarity and precision of results, including but not limited to blood collection technique (e.g., needle size or blood draw rate that impacts shear stress), sample centrifugation, timing of sample processing, sample freezing, thawing, and storage [102]. Generally speaking, EVs collected from fresh blood is considered more accurate and reflective true *in vivo* EV levels when compared with frozen samples [101, 103], but plasma frozen for only 24 hr may yield comparable counts when compared to fresh sample [104]. In either case, it is suggested that samples should be analyzed after the same “freezing period” [105] to enhance accuracy of sample analysis. Centrifugation speed crucially affects the type of EV population isolated. Most of the studies analyzing EVs in exercise interventions have utilized low centrifugation speeds (Tables 1 and 2, resp.). As different speeds are used, they have likely isolated different EV populations. In addition, they might have enriched for larger EVs and therefore used the term microparticles. However, this topic is still in debate and our work [10, 94] and that of others [11–13] indicates that we also see smaller EVs (e.g., exosomes, <100 nm) in these preparations, thereby making it difficult to distinguish between various types of EVs [11].

Conventional flow cytometry is the most commonly used technique for phenotyping and enumeration of EVs [106]. However, many older flow cytometer models limit the detection of smaller EVs, thereby contributing to potential gaps in our understanding of all subtypes of EVs [10]. Indeed, recent evidence suggests that while >80% of EVs are <500 nm, most conventional flow cytometers have a detection threshold greater than 500 nm, suggesting that a vast majority of EVs may not be quantified [107] with this technique. To address this discrepancy, an alternative approach has been developed, combining flow cytometry with imaging (called imaging flow cytometry). Erdbrügger et al. found that by adding imaging to flow cytometry, EVs can be clearly differentiated from the beads and cells, as well as debris. It also provides the advantage of confirming the presence of these vesicles based not only on fluorescence but also on scatter and morphology as well [108]. By detection of EV fluorescence only, even smaller EVs can be detected. The detection threshold is likely down to 100–200 nm. To date though, no prospective exercise research exists utilizing this approach to assess EV phenotypes. As interest in the role of EVs as mediators and markers of disease continues to grow, implementation of standardized EV approaches will be needed to elucidate the exact role of EVs in chronic disease. One approach to close this method gap is that future studies consider using established guidelines by the EV-TRACK Consortium to improve transparency in reporting EV research [106] and follow minimal experimental requirements for definition of EVs and their functions, as published by the International Society for Extracellular Vesicles [12]. Finally, implementation of these minimal experimental requirements described [12] is crucial in moving forward with functional studies combined with content analysis (genetic, proteomic, and metabolomics) in order to better advance our understanding preventing/treating chronic disease in relation to EVs.

## 7. Analysis of Smaller Extracellular Vesicles

Most of the studies discussed so far have used low centrifugation speeds to enrich for EVs, but likely analyzed a mix of large and smaller vesicles in their preparations. A few studies have focused on use of high centrifugation speed of 100,000 G to enrich for smaller EVs called exosomes. It is important to study all subtypes of EVs given that they play roles in immune modulation [109, 110], activating tissue repair [111], and angiogenesis as the following studies demonstrate. Interestingly, Fruhbeis et al. was one of them to report that cycling exercise increased smaller EVs (called exosomes, positive for Flot1, Hsp/Hsc70, and Int*α*IIb) to a greater extent when compared to treadmill exercise, but the rise in these smaller EVs (exosomes) remained elevated for a longer period of time into recovery with treadmill exercise [112]. The reason for these differential responses between treadmill and cycling exercise is not clear, but it might relate to the higher heart rate and eccentric muscle contraction associated with running. This would be consistent with prior work [65], suggesting that EVs are important for vascular repair and adaptation. Moreover, recent work from Safdar et al. has suggested that smaller EVs (exosomes) may be essential following endurance-oriented exercise as a means to treat metabolic disease [113]. This assertion is supported by evidence from Bei et al. who demonstrated that exercise-induced increases in circulating EVs enhanced the protective effects of endogenous EVs against cardiac ischemia/reperfusion injury [114]. These later findings are consistent with new work highlighting that exosomes play critical roles in interorgan crosstalk during exercise to regulate energy homeostasis [66]. Taken together, these preliminary data suggest more work is needed to characterize all subtypes of EVs, including smaller (exosomes) and larger (microparticle) EVs in people with T2D and CVD following different doses of exercise, with or without diet modification, to improve clinical practice for patient care.

## 8. Conclusion and Clinical Perspectives

The precise mechanism by which exercise lowers CVD is unclear, as only 40–50% of the reduction in CVD risk in subjects reporting >1500 kcal/week of exercise is attributed to nontraditional CVD risk factors [115]. EVs have emerged as novel markers of T2D and CVD that have potential functional and therapeutic benefit by transferring proteins, lipids, and nucleic acids. In fact, EV physiology appears critical towards the production of oxidative stress [54], inflammation [23], and/or physical contact/release of signaling molecules (i.e., miRNA) that modulate endothelial function [116]. Herein, we present evidence that suggests EVs represent a potentially novel mechanism by which exercise could fill a “cardio-protection risk gap.” Exercise may impact EVs by not only reducing substrates thought to drive EV functional responses but also altering the release of oxidative stress, inflammatory cytokines, and miRNA (Figure 1). Indeed, the acute effects of exercise on EVs are limited to endothelium-derived EVs (CD62E^+^, CD31^+^/CD42b^−^, CD144^+^) with little change or slight increases and few to no work on platelet- or leukocyte-derived EVs (Table 1). In contrast, exercise training appears to have more robust effects on decreasing endothelium-, platelet-, and leukocyte-derived EVs in men and women (Table 2). However, these studies are limited in that conventional flow cytometry has been used, thereby providing less sensitivity to detecting a variety of EV sizes (<500 nm) as well as distinguishing EVs from small cells/debris. Further work is needed using various tools including imaging or high-resolution flow cytometry, tunable resistive pulse sensing, or nanoparticle tracking device and electron microscopy before and after exercise interventions in order to ascertain a comprehensive EV profile in adults at risk for and with T2D or CVD. Knowledge of EV content and function may ultimately lead to improved patient care by enabling health care providers to provide bioengineered agents that mitigate “cargo” released from these EVs and/or deliver exercise-derived EVs as therapeutic options for optimization of T2D and CVD management.

## Figures and Tables

**Table 1:** Acute effects of exercise on circulating extracellular vesicles.

*Author*	*Exercise dose*	*Subtype EV*	*Preanalytical phase*	*Collection time*	*Response*
Harrison et al. [76]	Cycling for 90 min at 70% VO_2_peak followed by ten 1 min sprints interspersed with 1 min of recovery in young, recreationally active men.	CD31+/CD42b-	1600 g for 15 min at 4°C	Morning following exercise bout	No change

Mobius-Winkler et al. [63]	4 hr cycling at 70% IAT in young, healthy men.	CD42b^−^CD42b^−^/CD62E^+^	11,000*g* for 2 min	16 predefined time points during and after finishing cycling.	No change

Jenkins et al. [74]	Exercise at 70% of VO_2_peak until 598 kcal was expended in recreationally, healthy active men.	CD31^+^/CD42b^−^CD62E^+^	Double centrifugation of 1500*g* for 20 min at room temperature	(i) Pre-meal (ii) 1, 2, 3, and 4 hr postprandial	↓CD31^+^/CD42b^−^ ↓CD62E^+^ Unaffected by high-fat meal

Guiraud et al. [60]	Single session of HIIE: 15-second intervals at 100% of PPO and 15-second passive recovery intervals or isocaloric MICE in men with coronary heart disease.	CD31^+^ CD62E^+^ CD42b^−^ CD42b^+^	1500*g* for 15 min followed by a single centrifugation at 13,000*g* for 2 min	(i) 10 min pre-ex (ii) 20 min post-ex (iii) 24 hr post-ex (iv) 72 hr post-ex	No change

Ross et al. [62]	3 sets of 6 resistance exercises at 15 RM w/o rest in young, trained men.	CD144^+^ CD146^+^ CD105^+^	1500*g* for 15 min followed by 13,000*g* for 2 min	(i) Pre-ex (ii) 10 min post-ex (iii) 2 hr post-ex (iv) 24 hr post-ex	No change

Wahl et al. [67]	(1) HVT; 130 min at 55% PPO, (2) 4 × 4 min at 95% PPO, and (3) 4 × 30 sec all-out in healthy male, triathletes.	CD31^+^/CD42b^−^ CD31/CD42b CD14/CD16	1.861*g* for 10 min at 4°C	(i) Pre-ex (ii) Immediately post-ex (iii) 60 min post-ex (iv) 180 min post-ex	↓CD31^+^/CD42b^−^ ↓CD31/CD42b ↓CD14/CD16

Chanda et al. [61]	Acute strenuous exercise (treadmill running VO_2_peak) versus. moderate (75% HR_max_ for 45 min) in healthy females.	Total EVs CD41a	2500 rpm for 5 min at 4°C	(i) Pre-ex (ii) Immediately post-ex (iii) 45 min post-ex	↑Total EVs ↑CD41a Only after strenuous ex; No moderate ex change

Durrer et al. [72]	(1) HICE 20 min cycling at just above VT. (2) HIIE 10 × 1 min at 90% peak aerobic power in young, overweight, inactive males and females.	CD62E^+^ CD31^+^/CD42b^−^	Double centrifugation of 1500*g* for 15 min at room temperature	(i) Pre-ex (ii) Immediate post-ex (iii) Morning post-ex	↑CD62E^+^ (females only) No change CD31^+^/CD42b^−^ ↓CD62E^+^ (males only) ↓CD31^+^/CD42b^−^

Lansford et al. [69]	Acute bout at 60–75% VO_2_peak in healthy, active individuals	CD62E^+^, CD34^+^ CD31^+^/CD42b^−^	Double centrifugation of 1500*g* for 20 min at room temperature	(i) Pre-ex (ii) Post-ex	↑CD62E^+^ in men ↑CD34^+^ in women No change CD31^+^/CD42b^−^

Wilhelm et al. [65]	1 h of moderate- (46 ± 2% VO_2_peak) or heavy- (67 ± 2% VO_2_peak) intensity semirecumbent cycling in healthy, young men.	CD62E^+^ CD41^+^	17,500*g* for 1 hr at 4°C	(i) Pre-ex (ii) Post-ex	↑CD41^+^ following heavy exercise No CD62E^+^ change

Bei et al. [114]	Exercise stress test in middle-aged, overweight men and women.	CD63^+^	1000*g* for 10 min and 2500*g* for 15 min at room temperature	(i) Rest (ii) Peak-ex (iii) Recovery (15 min after completion	↑EV count

Whitham et al. [66]	1 hr (30 min at 55%, 20 min at 70%, and ~10 min at 80% of VO_2_peak) in healthy males.	ACTN4, ADAM10, ALIX, ANAX11, and CD81	20,000*g* for 2x at 60 min	(i) Pre-ex (ii) Exercise (iii) 4 hr post-ex	↑EV count

IAT: individual anaerobic threshold; HIIE: high-intensity interval exercise; MICE: moderate-intensity continuous exercise; RM: repetition max; HVT: high-volume training; PPO: peak power output; HICE: high-intensity continuous exercise; VT: ventilatory threshold.

**Table 2:** Chronic effects of exercise on circulating extracellular vesicles.

*Author (year)*	*Exercise dose*	*Subtype EV*	*Preanalytical phase*	*Collection time*	*Response*
La Vignera et al. [87]	150 min aerobic activity/wk for 3 mo in individuals with and without erectile dysfunction.	CD45^−^/CD34^−^/CD144^+^	Specifics not reported	(i) Baseline (ii) 3 mo	↓CD45^−^/CD34^−^/CD144^+^

Babbitt et al. [90]	24 wk aerobic training, 3x/wk, 40 min at 65% VO_2_peak in sedentary, middle-aged African American adults.	CD62E^+^	2000*g* for 20 min at 4°C	(i) Baseline (ii) 6 mo	↓CD62E^+^

Kretzschmar et al. [89]	6 mo aerobic training, 3x/wk, 40 min at 65% VO_2_peak in pre- and postmenopausal African American women.	CD62E^+^ CD31^+^/CD42b^−^	Double centrifugation at 1500*g* for 20 min at 24°C	(i) Baseline (ii) 6 mo	↓CD62E^+^ ↓CD31^+^/CD42b^−^ in the premenopausal group only

Bruyndonckx et al. [86]	10 months of 3 supervised sessions/wk combined with diet intervention in obese children between the ages of 12 and 18.	CD31^+^/CD42b^−^	Double centrifugation of 1525*g* for 20 min	(i) Baseline (ii) 5 mo (iii) 10 mo	↓CD31^+^/CD42b^−^

Kim et al. [88]	3 days/wk for 6 mo of 40 min at 65% of predicted HR_peak_ in adults with prehypertension.	CD31^+^/CD42b^−^, CD62E^−^	Double centrifugation of 1500*g* for 20 min	(i) Baseline (ii) 6 mo	↓CD31^+^/CD42a^−^ ↓CD62E^+^

Pitha et al. [91]	6 mo of supervised training on a cycle ergometer in renal transplant recipients.	CD34^+^ CD45^+^	ELISA	(i) Baseline (ii) Median of 6 mo	No change

Van Craenenbroeck et al. [95]	12 wk, 3x/wk aerobic continuous training (70–75% HR_peak_) or aerobic interval training (four 4 min intervals 90–95% of HR_peak_ with 3 min recovery at 50–70% of HR_peak_) adults with coronary artery disease.	CD31^+^/CD42b^−^	Double centrifugation at 1550*g*	(i) Baseline (ii) 12 wk	No change

**Table 3:** Most commonly used extracellular vesicles.

EV origin	Surface markers
Endothelium	**CD31 ** ^**+**^ ** /CD41 ** ^**−**^ ** (PECAM ** ^**+**^ ** /ITGA2B ** ^**−**^ ** )** CD31^+^/CD42^−^ (PECAM^+^/GPIb^−^) **CD31 (PECAM (platelet cell adhesion molecule))** CD144 (VE cadherin (vascular endothelial cadherin)) CD146 (MCAM (melanoma cell adhesion molecule)) CD105 (endoglin) CD106 (VCAM (vascular cell adhesion molecule)) **CD62E (E-selectin (endothelial-selectin))**

Platelet	**CD41 (ITGA2B (integrin alpha 2b))** CD42 (GPIb (glycoprotein Ib)) CD31 (PECAM (platelet cell adhesion molecule))

Leukocyte	CD45 (PTPRC (protein tyrosine phosphate receptor type C)) CD11b (ITGAM (integrin alpha M)) CD14 (coreceptor of lipopolysaccharide) CD16 (on surface of neutrophils, monocytes, and macrophages) CD62L (L-selectin (leukocyte selectin))

Red blood cell	CD235 (glycophorin A)

## Abbreviations

EVs: Extracellular vesicles
T2D: Type 2 diabetes
CVD: Cardiovascular disease.
